# Supplementary material for: Effect of acute kidney injury on the patients with hepatocellular carcinoma undergoing transarterial chemoembolization
Source: PLoS One. 2020 Dec 14;15(12):e0243780. doi: 10.1371/journal.pone.0243780 (PMC7735598; doi:10.1371/journal.pone.0243780)
Supplement: S1 File — (DOCX) [file pone.0243780.s001.docx]

**Summary of clinical variables in the dataset**

| Variables | Age | Platelet count | PT_INR | albumin | Total bilirubin | AST | ALT | AFP | MELD | SCr_baseline | SCr_D1 | SCr_Mo2 | Scr_Mo4 |
| --- | --- | --- | --- | --- | --- | --- | --- | --- | --- | --- | --- | --- | --- |
| Mean | 60.94 | 156.79 | 1.1171 | 3.815 | 1.0347 | 74.68 | 51.30 | 1268.9571 | 8.36 | .883 | .864 | .917 | 1.049 |
| Standard error of mean | .571 | 4.364 | .00743 | .0219 | .02121 | 5.924 | 3.595 | 170.40923 | .104 | .0101 | .0116 | .0191 | .0433 |
| Median | 61.00 | 140.00 | 1.1000 | 3.800 | 1.0000 | 52.00 | 39.00 | 23.2800 | 8.00 | .900 | .900 | .900 | .900 |
| Standard deviation | 10.641 | 81.287 | .13840 | .4078 | .39505 | 110.357 | 66.960 | 3174.37227 | 1.938 | .1886 | .2140 | .3493 | .7736 |
| Variance | 113.230 | 6607.622 | .019 | .166 | .156 | 12178.571 | 4483.643 | 10076639.312 | 3.757 | .036 | .046 | .122 | .598 |
| Minimum | 29 | 30 | .82 | 2.8 | .20 | 14 | 7 | 0 | 3 | .4 | .3 | .2 | .2 |
| Maximum | 88 | 667 | 2.20 | 4.8 | 2.49 | 1753 | 957 | 12500.00 | 22 | 1.5 | 1.7 | 4.0 | 8.2 |
| 25 percentiles | 53.00 | 100.00 | 1.0300 | 3.500 | .7600 | 37.00 | 24.00 | 4.2900 | 7.00 | .800 | .700 | .800 | .800 |
| 75 percentiles | 69.00 | 196.00 | 1.1900 | 4.100 | 1.2300 | 84.00 | 59.00 | 271.1000 | 9.00 | 1.000 | 1.000 | 1.000 | 1.000 |

*Abbreviation: PT_INR, prothrombin time international normalized ratio; AST, aspartate aminotransferase; ALT, alanine aminotransferase; AFP, alpha-fetoprotein; MELD, model for end-stage liver disease; SCr, serum creatinine; SCr_baseline, serum creatinine at pre-TACE; SCr_D1, serum creatinine at 1 day after TACE; SCr_Mo2, serum creatinine at 2 months after TACE; SCr_Mo4, serum creatinine at 4 months after TACE.
