# Supplementary material for: Effect of acute kidney injury on the patients with hepatocellular carcinoma undergoing transarterial chemoembolization
Source: PLoS One. 2020 Dec 14;15(12):e0243780. doi: 10.1371/journal.pone.0243780 (PMC7735598; doi:10.1371/journal.pone.0243780)
Supplement: Attachment — Submitted filename: Respone to reviewers_20201021.docx [file pone.0243780.s002.docx]

**Editor’s comments:**
1. Please ensure that your manuscript meets PLOS ONE's style requirements, including those for file naming. The PLOS ONE style templates can be found at

https://journals.plos.org/plosone/s/file?id=wjVg/PLOSOne_formatting_sample_main_body.pdf and

https://journals.plos.org/plosone/s/file?id=ba62/PLOSOne_formatting_sample_title_authors_affiliations.pdf

: As you commented, we revised our manuscript according to PLOS ONE's style requirements

2. During our internal review, we noticed you have overlapping text with an abstract of your work published here: https://aasldpubs.onlinelibrary.wiley.com/doi/10.1002/hep.30941#

PLOS ONE cannot (re)publish material without sufficient permission from the original copyright holder to publish under a CC BY license. Please provide proof that the owner of the content (a) has given you written permission to use it, and (b) has approved of the CC BY license being applied to their content. You may have the following form completed by the owner as proof: https://journals.plos.org/plosone/s/file?id=7c09/content-permission-form.pdf. Alternatively, you may electronically request permissions electronically from the copyright owner and send us proof of approval, as long as the approval clearly shows that the owner has approved of the CC BY license being applied to their content. Please see https://journals.plos.org/plosone/s/licenses-and-copyright for more information

: As you commented, the abstract of this study was presented in ‘The Liver Meeting 2019’, organized American Association for the study of Liver Diseases (AASLD) and held from Nov 08-12, 2019 at Boston, Massachusetts, United States of America. The abstract was published in Hepatology 2019;70(S1):188-1382 (John Willey and Sons).

The policy of ‘Copyright Transfer Agreements (CTA) for Conference Abstracts’ in Wiley is as follows (<https://authorservices.wiley.com/author-resources/Journal-Authors/licensing/licensing-info-faqs.html>).

Conference abstracts: We do not require a signed Agreement to publish abstracts of material submitted for poster sessions and presentations at conferences or meetings.

According to the policy of Wiley, we think it is not necessary to get the permission of copyright for the abstract of my manuscript.

3. In your Methods section, please provide additional information about the participants and recruitment method and the demographic details of your participants. Please ensure you have provided sufficient details to replicate the analyses such as:

a) the recruitment date range (month and year),

b) a description of any inclusion/exclusion criteria that were applied to participant recruitment,

c) a statement as to whether your sample can be considered representative of a larger population,

d) a description of how participants were recruited, and

e) a reference for the historical cohort of Kangbuk Samsung Hospital

: We appreciate your valuable comments. According to your comments, we added below contents in the ‘Method’ section in the revised manuscript.

a) the recruitment date range (month and year),

: The recruitment date range of the study was from January 2000 to December 2014. We revised this content as follows.

(page 4, line 9)

“…… TACE as an initial treatment for HCC January 2000 - December 2014.”.

b) a description of any inclusion/exclusion criteria that were applied to participant recruitment,

: According to your comment, we added the inclusion and exclusion criteria in this study. We revised this content as follows.

(page 4, line 9)

“A total of 547 HCC patients underwent TACE as an initial treatment for HCC January 2000 – December 2014. We excluded 200 patients with the following reasons: (i) Child-Pugh class B or C (n=184); (ii) no information of serum creatinine (SCr) level during follow-up (n=10); (iii) follow-up loss within 1 month (n=2); (iv) pre-TACE SCr >1.5 mg/dL (n=4). Finally, a total of 347 HCC patients with Child-Pugh class A and pre-TACE SCr ≤1.5 mg/dL undergoing TACE as an initial therapy were analyzed.”

c) a statement as to whether your sample can be considered representative of a larger population,

: Thank you for your comment. We think the enrolled patients of this study could be considered representative of a larger population because the study was conducted based on the consecutive patients treated with TACE for HCC between January 2000 and December 2014. We added this content as follows in the ‘Discussion’ section of the revised manuscript.

(page 19, line 7)

“Also, we think the enrolled patients of this study could be considered representative of a larger population because the study was conducted based on the consecutive patients treated with TACE for HCC between January 2000 and December 2014.”

d) a description of how participants were recruited, and

e) a reference for the historical cohort of Kangbuk Samsung Hospital

: Thank you for your comment. This study was conducted based on a historical cohort of Kangbuk Samsung Hospital. The reference was added in the method section of the revised manuscript (Journal of Gastroenterology and Hepatology 2018;33:1397–1406). This historical cohort enrolled the consecutive patients with HCC for TACE as an initial treatment between January 2000 and December 2014. We revised this content as follows.

(page 4, line 8)

“This study was conducted based on a historical cohort of Kangbuk Samsung Hospital, Seoul, Korea [15]. A total of 547 HCC patients underwent TACE as an initial treatment for HCC January 2000 – December 2014.”

4. Please provide additional details regarding participant consent. In the ethics statement in the Methods and online submission information, please ensure that you have specified (1) whether consent was obtained, (2) whether consent was informed, and (3) what type you obtained (for instance, written or verbal, and if verbal, how it was documented and witnessed). If your study included minors, state whether you obtained consent from parents or guardians. If the need for consent was waived by the ethics committee, please include this information.

If you are reporting a retrospective study of medical records or archived samples, please ensure that you have discussed whether all data were fully anonymized before you accessed them and/or whether the IRB or ethics committee waived the requirement for informed consent. If patients provided informed written consent to have data from their medical records used in research, please include this information.

For additional information about PLOS ONE ethical requirements for human subjects research, please refer to <http://journals.plos.org/plosone/s/submission-guidelines#loc-human-subjects-research>.

: Thank you for your comment. This study was conducted in the retrospective manner using a historical cohort. Therefore, the requirement for informed consent was waived. All data were fully anonymized before the authors accessed them and/or whether the IRB waived the requirement for informed consent. We revised this content as follows.

(page 4, line 15)

“This study was conducted in the retrospective manner using a historical cohort. Therefore, the requirement for informed consent was waived. All data were fully anonymized before the authors accessed them and/or whether the institutional review board (IRB) waived the requirement for informed consent. The study was approved by the IRB of Kangbuk Samsung Hospital (IRB No. 2015-08-042).”

5. Thank you for stating the following financial disclosure:

"NO"

At this time, please address the following queries:

a.	Please clarify the sources of funding (financial or material support) for your study. List the grants or organizations that supported your study, including funding received from your institution.

b.	State what role the funders took in the study. If the funders had no role in your study, please state: “The funders had no role in study design, data collection and analysis, decision to publish, or preparation of the manuscript.”

c.	If any authors received a salary from any of your funders, please state which authors and which funders.

d.	If you did not receive any funding for this study, please state: “The authors received no specific funding for this work.”

: According to your comment, we addressed “The authors received no specific funding for this work.”.

6. Thank you for stating the following in your Competing Interests section:

"No"

Please complete your Competing Interests on the online submission form to state any Competing Interests. If you have no competing interests, please state "The authors have declared that no competing interests exist.", as detailed online in our guide for authors at http://journals.plos.org/plosone/s/submit-now

This information should be included in your cover letter; we will change the online submission form on your behalf.

: According to your comment, we addressed "The authors have declared that no competing interests exist.” in cover letter and changed this information on the online submission.

7. PLOS requires an ORCID iD for the corresponding author in Editorial Manager on papers submitted after December 6th, 2016. Please ensure that you have an ORCID iD and that it is validated in Editorial Manager. To do this, go to ‘Update my Information’ (in the upper left-hand corner of the main menu), and click on the Fetch/Validate link next to the ORCID field. This will take you to the ORCID site and allow you to create a new iD or authenticate a pre-existing iD in Editorial Manager. Please see the following video for instructions on linking an ORCID iD to your Editorial Manager account: <https://www.youtube.com/watch?v=_xcclfuvtxQ>

: According to your comment, the corresponding author validated the ORCID iD in Editorial Manager (ORCID iD: 0000-0002-7215-7715).

**Reviewer’s comments**

**Reviewer #1**

The authors concluded that AKI is a crucial prognostic factor for overall survival in HCC patients undergoing TACE. Appropriately written article on a recently focused issue. However, this article had a couple of critical points to be addressed, as follows:

#1. BCLC stage was significantly associated with AKI development. It should be better to assess the multicollinearity among predictor variables in Cox regression model.

: We appreciate your valuable comments. According to your comment, we performed the multivariable analysis considering multicollinearity in the logistic and Cox regression models. The multivariable analysis was performed as follows in reference to ‘Multivariable Analysis: A Practical Guide for Clinicians and Public Health Researchers 3rd edition’ (2011; Mitchell H. Katz: Cambridge University Press). First, we included in the multivariable analysis any variable that was associated with the outcome at a P-value of <0.10, regardless of whether or not the variable was associated with the risk factor. Second, for multivariable logistic regression analysis, the subjects of analysis should have at least ten outcomes for each independent variable in the multivariable model. Third, to consider the multicollinearity of the variables, the methods of variable selection such as forward selection or backward elimination are used in multivariable analysis model. We used the forward conditional stepwise method (in SPSS 18.0) in this study.

Considering the above-mentioned points, we performed the multivariable logistic and Cox regression analyses in this study. First, we analyzed the univariable and multivariable logistic regression models to assess the risk factors for AKI development after TACE (Table 3). The univariable analysis showed that AKI development after TACE is associated with platelet count, serum AFP ≥100 ng/mL, BCLC stage C, use of cisplatin as chemoembolization agents, presence of ascites and sessions of TACE ≥2. In this study, patients with AKI were reported 37 patients. Therefore, we selected 4 variables in multivariate analysis (37 patients/10 = 4 variables). We performed the multivariable analysis with the forward conditional stepwise method to consider multicollinearity and by four variables (platelet count, BCLC stage C, use of cisplatin as chemoembolization agents, presence of ascites) which were selected by univariable analysis. Finally, BCLC stage C, and presence of ascites were the independent risk factors for significant fibrosis after multivariable analysis using four variables.

Second, we analyzed the univariable and multivariable Cox regression models to assess the risk factors for overall survial after TACE (Table 4). The univariable analysis showed that overall survial after TACE is associated with platelet count, serum albumin level, serum AFP level, maximal tumor size, number of tumor, bilobar location of tumor, portal vein invasion, BCLC stage, use of cisplatin as chemoembolization agents, presence of ascites, and AKI stage. In this study, death occurred in 109 patients. Therefore, we selected 11 variables in multivariate analysis 109 patients/10 = 11 variables). We performed the multivariable analysis with the forward conditional stepwise method to consider multicollinearity and by all eleven variables which were selected by univariable analysis. Finally, serum albumin level, BCLC stage, bilobar location of tumor and AKI stage were the independent risk factors for significant fibrosis after multivariable analysis using eleven variables. We added these contents in the ‘method’ section of the revised manuscript.

(page 7, line 2)

“Multivariable analysis in logistic and Cox regression models was done in the following manners. First, we included in the multivariable analysis any variable that was associated with the outcome at a P-value of <0.10, regardless of whether or not the variable was associated with the risk factor. Second, for multivariable regression analysis, the subjects of analysis should have at least ten outcomes for each independent variable in the multivariable model. Third, multivariable analysis was performed using a forward conditional stepwise procedure to avoid multicollinearity.”

#1. There is a typo in the sentence below (page 7, baseline characteristics section):

BCLC stages were non-AKI in 23 patients (6%), stage A in 124 patients (36%), stage B in 93 patients (27%), and stage C in 107 patients (31%).

: According to your comment, we corrected a typo in the revised manuscript.

(page 8, line 3)

“BCLC stages were stage 0 in 23 patients (6%), stage A in 124 patients (36%), stage B in 93 patients (27%), and stage C in 107 patients (31%).”

#2. It is difficult to check the back part of Table 2. Please modify the Table 2.

: According to your comment, we modified Table 2 in the revised manuscript.

#3. Please, upload modified figures with improved resolution.

: According to your comment, we modified the figures with improved resolution.

**Reviewer #2**

Dr Sohn has studied the effect of AKI on HCC patients undergoing TACE. The findings are important and have a clinical impact. However, I have several minor comments and requests before being accepted.

1. Please show the effect AKI on survival based on subgroup analysis according to 1) BCLC stage 0/A and BCLC BC, 2) albumin level with cut off 3.5 and 3) presence of ascites as shown in the results.

: We appreciate your valuable comments. As you commented, we performed subgroup analysis for the effect of AKI on overall survival according to BCLC stage, serum albumin level and presence of ascites. First, we evaluated the effect of AKI stage on overall survival of HCC patients undergoing TACE according to BCLC stage 0/A vs. BCLC stage B/C. In a subgroup of BCLC stage 0/A, overall survival was significantly different according to AKI stage (*p*<0.001). The 12-month, 24-month, 48-month, 72-month, 96-month, and 120-month survival rates of patients were 99.3%, 92.5%, 80.8%, 75.6%, 72.0%, and 72.0%, respectively, in patients with non-AKI; and 0%, 0%, 0%, 0%, 0%, and 0%, respectively, in patients with AKI stage 2 or 3. In a subgroup of BCLC stage B/C, overall survival was significantly different according to AKI stage (*p*<0.001). The 12-month, 24-month, 48-month, 72-month, 96-month, and 120-month survival rates of patients were 78.8%, 72.7%, 60.2%, 75.6%, 37.2%, and 28.3%, respectively, in patients with non-AKI; and 18.8%, 0%, 0%, 0%, 0%, and 0%, respectively, in patients with AKI stage 1; and 0%, 0%, 0%, 0%, 0%, and 0%, respectively, in patients with AKI stage 2 or 3.

Second, we evaluated the effect of AKI stage on overall survival of HCC patients undergoing TACE according to serum albumin ≤3.5 g/dL vs. >3.5 g/dL. In a subgroup of serum albumin >3.5 g/dL, overall survival was significantly different according to AKI stage (*p*<0.001). The 12-month, 24-month, 48-month, 72-month, 96-month, and 120-month survival rates of patients were 89.4%, 83.3%, 74.9%, 64.7%, 59.5%, and 59.5%, respectively, in patients with non-AKI; and 0%, 0%, 0%, 0%, 0%, and 0%, respectively, in patients with AKI stage 1; and 47.1%, 0%, 0%, 0%, 0%, and 0%, respectively, in patients with AKI stage 2 or 3. In a subgroup of serum albumin ≤3.5 g/dL, overall survival was significantly different according to AKI stage (*p*<0.001). The 12-month, 24-month, 48-month, 72-month, 96-month, and 120-month survival rates of patients were 88.1%, 80.6%, 58.4%, 37.0%, 37.0%, and 24.7%, respectively, in patients with non-AKI; and 66.7%, 0%, 0%, 0%, 0%, and 0%, respectively, in patients with AKI stage 1; and 0%, 0%, 0%, 0%, 0%, and 0%, respectively, in patients with AKI stage 2 or 3.

Finally, we evaluated the effect of AKI stage on overall survival of HCC patients undergoing TACE according to presence of ascites. In a subgroup of patients without ascites, overall survival was significantly different according to AKI stage (*p*<0.001). The 12-month, 24-month, 48-month, 72-month, 96-month, and 120-month survival rates of patients were 89.9%, 83.7%, 72.2%, 60.2%, 56.0%, and 52.0%, respectively, in patients with non-AKI; and 50%, 0%, 0%, 0%, 0%, and 0%, respectively, in patients with AKI stage 1; and 0%, 0%, 0%, 0%, 0%, and 0%, respectively, in patients with AKI stage 2 or 3. In a subgroup of patients with ascites, overall survival was significantly different according to AKI stage (*p*<0.001). The 12-month, 24-month, 48-month, 72-month, 96-month, and 120-month survival rates of patients were 81.7%, 72.6%, 57.2%, 22.9%, 22.9%, and 22.9%, respectively, in patients with non-AKI; and 0%, 0%, 0%, 0%, 0%, and 0%, respectively, in patients with AKI stage 1; and 0%, 0%, 0%, 0%, 0%, and 0%, respectively, in patients with AKI stage 2 or 3. Therefore,
AKI stage is an independent risk factor for overall survival of HCC patients undergoing TACE regardless of BCLC tumor stage, serum albumin level and presence of ascites. We added these findings in the ‘Result’ section of the revised manuscript.

(page 14-16, line 24)

“We performed subgroup analysis for the effect of AKI on overall survival according to BCLC stage, serum albumin level and presence of ascites. First, we evaluated the effect of AKI stage on overall survival of HCC patients undergoing TACE according to BCLC stage 0/A vs. BCLC stage B/C. In a subgroup of BCLC stage 0/A, overall survival was significantly different according to AKI stage (*p*<0.001). The 12-month, 24-month, 48-month, 72-month, 96-month, and 120-month survival rates of patients were 99.3%, 92.5%, 80.8%, 75.6%, 72.0%, and 72.0%, respectively, in patients with non-AKI; and 0%, 0%, 0%, 0%, 0%, and 0%, respectively, in patients with AKI stage 2 or 3. In a subgroup of BCLC stage B/C, overall survival was significantly different according to AKI stage (*p*<0.001). The 12-month, 24-month, 48-month, 72-month, 96-month, and 120-month survival rates of patients were 78.8%, 72.7%, 60.2%, 75.6%, 37.2%, and 28.3%, respectively, in patients with non-AKI; and 18.8%, 0%, 0%, 0%, 0%, and 0%, respectively, in patients with AKI stage 1; and 0%, 0%, 0%, 0%, 0%, and 0%, respectively, in patients with AKI stage 2 or 3. Second, we evaluated the effect of AKI stage on overall survival of HCC patients undergoing TACE according to serum albumin ≤3.5 g/dL vs. >3.5 g/dL. In a subgroup of serum albumin >3.5 g/dL, overall survival was significantly different according to AKI stage (*p*<0.001). The 12-month, 24-month, 48-month, 72-month, 96-month, and 120-month survival rates of patients were 89.4%, 83.3%, 74.9%, 64.7%, 59.5%, and 59.5%, respectively, in patients with non-AKI; and 0%, 0%, 0%, 0%, 0%, and 0%, respectively, in patients with AKI stage 1; and 47.1%, 0%, 0%, 0%, 0%, and 0%, respectively, in patients with AKI stage 2 or 3. In a subgroup of serum albumin ≤3.5 g/dL, overall survival was significantly different according to AKI stage (*p*<0.001). The 12-month, 24-month, 48-month, 72-month, 96-month, and 120-month survival rates of patients were 88.1%, 80.6%, 58.4%, 37.0%, 37.0%, and 24.7%, respectively, in patients with non-AKI; and 66.7%, 0%, 0%, 0%, 0%, and 0%, respectively, in patients with AKI stage 1; and 0%, 0%, 0%, 0%, 0%, and 0%, respectively, in patients with AKI stage 2 or 3. Finally, we evaluated the effect of AKI stage on overall survival of HCC patients undergoing TACE according to presence of ascites. In a subgroup of patients without ascites, overall survival was significantly different according to AKI stage (*p*<0.001). The 12-month, 24-month, 48-month, 72-month, 96-month, and 120-month survival rates of patients were 89.9%, 83.7%, 72.2%, 60.2%, 56.0%, and 52.0%, respectively, in patients with non-AKI; and 50%, 0%, 0%, 0%, 0%, and 0%, respectively, in patients with AKI stage 1; and 0%, 0%, 0%, 0%, 0%, and 0%, respectively, in patients with AKI stage 2 or 3. In a subgroup of patients with ascites, overall survival was significantly different according to AKI stage (*p*<0.001). The 12-month, 24-month, 48-month, 72-month, 96-month, and 120-month survival rates of patients were 81.7%, 72.6%, 57.2%, 22.9%, 22.9%, and 22.9%, respectively, in patients with non-AKI; and 0%, 0%, 0%, 0%, 0%, and 0%, respectively, in patients with AKI stage 1; and 0%, 0%, 0%, 0%, 0%, and 0%, respectively, in patients with AKI stage 2 or 3. Therefore, AKI stage is an independent risk factor for overall survival of HCC patients undergoing TACE regardless of BCLC tumor stage, serum albumin level and presence of ascites.”

2. Have you ruled out the possibility of sepsis, the use of nephrotoxic drug or increasing ascites that could have been the cause for AKI?

: Thank you for your comment. We re-analyzed possible causes for AKI development after TACE. Of 37 patients with AKI development, 8 patients had experienced increasing ascites. Use of ACE inhibitor or ARB, and beta-blocker was observed in 6 patients, and 6 patients, respectively. Bacterial infection after TACE was observed in 5 patients (spontaneous bacterial peritonitis in 4 patients; pneumonia in 1 patient). We added these findings in the ‘Result’ section of the revised manuscript.

(page 9, line 21)

“Possible causes for AKI development after TACE were analyzed. Of 37 patients with AKI development, 8 patients had experienced increasing ascites. Use of ACE inhibitor or ARB, and beta-blocker was observed in 6 patients, and 6 patients, respectively. Bacterial infection after TACE was observed in 5 patients (spontaneous bacterial peritonitis in 4 patients; pneumonia in 1 patient).”

3. The cisplatin based treatment is associated with occurrence of AKI compared to doxorubicin. However, have you observed the volume of contrast medium which could have affected AKI?

: Thank you for your comment. That point is important in this study. However, we could not evaluate the volume of contrast medium because the study was conducted in the retrospective manner using a historical cohort. Therefore, this study could not assess the effect of the volume of contrast medium on AKI development after TACE for HCC. We described this limitation in the discussion section of the revised manuscript

(page 19, line 2)

“Finally, we could not evaluate the volume of contrast medium because the study was conducted in the retrospective manner using a historical cohort. Therefore, this study could not assess the effect of the volume of contrast medium on AKI development after TACE for HCC.”

Minor comment: Final column of table 2 does not fit into page and was unreadable.

: According to your comment, we modified Table 2 in the revised manuscript.
